# Routine thyroidectomy with total laryngectomy: Is it really indicated? A randomized controlled trial

**DOI:** 10.1016/j.amsu.2022.103309

**Published:** 2022-01-31

**Authors:** May El-Sebai Ali, Hisham Atef Ebada, Mahmoud Abd El-Shaheed, Ahmed Musaad AbdElFattah, EL Sharawy Kamal

**Affiliations:** a Department of Otorhinolaryngology-Faculty of Medicine-Mansoura University, Mansoura, Egypt; b Department of Radiology -Faculty of Medicine-Mansoura University, Mansoura, Egypt

**Keywords:** Thyroidectomy, Laryngectomy, Hypothyroidism, Recurrence

## Abstract

**Background:**

We investigated the incidence of thyroid gland invasion in patients with advanced laryngeal carcinoma who were treated with total laryngectomy, also the impact of different preoperative and intraoperative predictors on thyroid gland invasion. Moreover, the impact of thyroid gland preservation on the locoregional tumor control and the recurrence rates after surgery were investigated.

**Materials and methods:**

This study was conducted over 5 years on 100 patients with advanced laryngeal carcinoma who underwent total laryngectomy. The adopted protocol in our hospital is to perform an ipsilateral thyroid lobectomy if there is subglottic extension, thyroid or cricoid cartilage invasion or true invasion of the thyroid gland. The patients of the study were divided into thyroid sparing and thyroid sacrificing groups. The two groups were compared in terms of demographic data, tumor characteristics, incidence of postoperative hypothyroidism and tumor recurrence.

**Results:**

There was no significant difference between groups regarding the tumor profile. Regarding local tumor spread, the only two subsites that showed significant difference is anterior commissure and thyroid cartilage invasion. In the thyroid sacrificing group, invasion of the thyroid gland was proved histopathologically in only one patient. Postoperatively, the incidence of hypothyroidism was significantly higher in the thyroid sacrificing group. However, there was no statistically significant difference between the two groups regarding the incidence of tumor recurrence.

**Conclusion:**

The incidence of thyroid gland invasion by an advanced laryngeal carcinoma is low. Preservation of the thyroid gland during laryngectomy to reduce the risk of thyroid dysfunction does not affect the oncological control.

## Highlights

• Thyroid gland invasion incidence in advanced laryngeal carcinoma is low.
• Thyroid gland Preservation during laryngectomy does not affect the oncological control.
• There was no statistically significant difference between the thyroid sparing and thyroid sacrificing group regarding the incidence of recurrence. Due toding author”. Due toding author".

## 1 Introduction

Management of the thyroid gland during laryngectomy is still controversial [1]. The American National Cancer Network (National Comprehensive Cancer Network) recommended ipsilateral hemithyroidectomy with laryngectomy in advanced stage laryngeal carcinomas [2]. Thyroidectomy is performed for possibility of its invasion by the tumor [3]. Carcinoma of the larynx can invade the thyroid gland by direct extension or less frequently can spread indirectly by lymphatic or vascular channels [4].

However, thyroid gland invasion by advanced (T3 and T4) carcinoma of the larynx is uncommon with overall incidence of about 5–12.6% [5,6]. Furthermore, patients who undergo thyroidectomy may suffer from thyroid and parathyroid dysfunction. It requires life-long monitoring, replacement therapies and follows up [7,8]. Adjuvant radiotherapy is used in the majority of these patients which increases the risk of hypothyroidism [9,10]. Hypothyroidism can be insidious, and may lead to impaired wound healing, fistula formation, decreased cardiac function, systemic morbidities and depression [10,11].

It is still controversial whether it is appropriate to do thyroidectomy in all advanced laryngeal carcinoma patients, or in only selected cases [1]. Some clinical trials indicate that thyroidectomy is only suggested with laryngectomy in the presence of thyroid gland invasion, subglottic extension, thyroid cartilage invasion or involvement of the pyriform sinus apex [5,6,9,12].

*Objectives*: To investigate the incidence of thyroid gland invasion in patient with advanced laryngeal carcinoma and the impact of different predictors on thyroid gland invasion. The impact of thyroid gland preservation on the locoregional tumor control was investigated.

## 2 Patients and methods

This randomized controlled trial was conducted over 5 years (from September 2014 to September 2019), in the Otolaryngology department, Mansoura University Hospitals, Egypt. It was approved by the … … Faculty of Medicine Institutional Research Board (MFM-IRB: MD/16.05.94). Informed written consents were obtained from all the patients included in the study.

This work included 112 consecutive patients with advanced T3 and T4 laryngeal carcinoma who underwent total laryngectomy and accepted to enroll in the trial. Twelve patients were excluded from the study due to past history of thyroidectomy prior to laryngectomy (n = 3), preoperative radiotherapy (n = 7) or lost patients in the follow up (n = 2). The remaining 100 patients were evaluated.

All patients were subjected for history taking regarding the presenting symptoms and any associated upper aerodigestive tract symptoms. Additional information was gathered including exposure to risk factors for laryngeal cancer (primarily tobacco and alcohol), medications, and medical comorbidities.

Head and neck examination was performed to assess the location and extent of the primary tumor and palpating for neck nodes. Flexible nasal laryngoscope was done to assess the site of the primary tumor, involved subsites, extension to adjacent structures, true vocal fold mobility, and patency of the airway.

Imaging was performed before operative biopsy to obtain images before potential edema and distortion from biopsying and manipulation of the larynx. A contrast-enhanced CT was performed for all patients. Scans were obtained from the base of the tongue to the root of the neck using 2–3 mm thick sections. Intravenous contrast is especially important, because the primary tumor usually enhances with contrast, thus improving its visualization. Besides, the contrast will differentiate between nodal disease and blood vessels in the neck. MRI was performed as a complementary assessment in cases where cartilage infiltration or extra-laryngeal spread was suspected from the CT scan.

Laryngeal tumors were classified according to primary subsite as either supraglottic, glottic, subglottic or transglottic. Transglottic tumors represent tumors that involved all three subsites and/or those that crossed the laryngeal ventricle in a vertical direction. Subglottic extension was defined as any tumor that involved the subglottis either primarily or by extension from another site.

According to TNM staging, the American Joint Committee on cancer Staging Manual, 7th Edition, 2010 [13] was applied to tumor, node and metastasis. Histological grading had been performed according to the World Health Organization (WHO) criteria: well differentiated (G1), moderately differentiated (G2), and poorly differentiated (G3).

Total laryngectomy was performed for all patients. The adopted protocol in our hospital is to perform an ipsilateral thyroid lobectomy if there is subglottic extension, thyroid or cricoid cartilage invasion or true invasion of the thyroid gland. Total thyroidectomy is performed in bilateral laryngeal carcinoma with presence of one or more of the aforementioned variables. On the other hand, thyroid gland is preserved in absence of those factors.

The levels of thyroid stimulating hormone (TSH), as well as free thyroxine (FT4) were assayed by electrochemiluminescence immunoassay preoperatively, then periodically every 2 weeks in the first 3 months postoperatively, and subsequently every 6 months. Patients were stated to have thyroid dysfunction when the TSH level exceeded 10.0 mIU/L.

Accordingly, the patients of the study were divided into two groups: thyroid sparing group and thyroid sacrificing group (lobectomy or total thyroidectomy). The two groups were compared in terms of demographic data, smoking, alcohol consumption, tumor characteristics (location, stage, extension, histological type and grade of differentiation), incidence of postoperative hypothyroidism and tumor recurrence after surgery (see Fig. 1).

**Flow chart 1:**
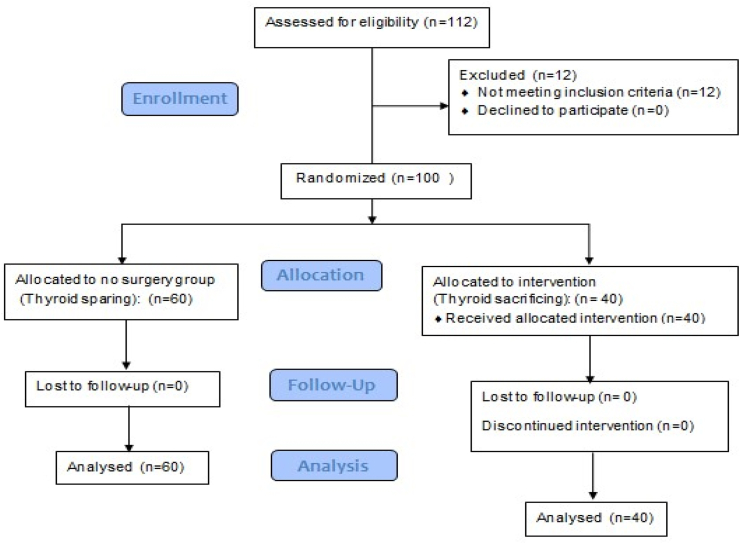
Participant flow.

In the thyroid sacrificing group, whole-organ total laryngectomies with thyroidectomies were sectioned, stained and examined by the head and neck pathology team to detect any thyroid gland invasion by the laryngeal carcinoma. The follow up period ranged from 6 months to 66 months (median 34.5).

*Inclusion Criteria:* Patients with pathological proven biopsy 1ry laryngeal Scc and in whom the treatment modality included total laryngectomy.

*Exclusion Criteria:* Patients with a pathological diagnosis other than primary laryngeal cancer, Primary thyroid cancer or previously treated with chemoradiation.

*Randomization:* The randomization was done as follows: when the first patient fulfilled the inclusion criteria, he was introduced into the thyroid sparing surgery group. The second patient then was introduced into the thyroid sacrificing group, the third into the first group and so on (simple randomization by flipping the coin).

Registration of research.

Research registry UIN: researchregistry7438. At the website: https://www.researchregistry.com/browse-heregistry#home/?view_2_page=2.

This work has been reported in line with the CONSORT criteria [14].

*Statistical analysis*: Data were analyzed using the Statistical Package of Social Science (SPSS) program for Windows (Standard version 21). Qualitative data were described using numbers and percentages. Study power is 0.8. Association between categorical variables was tested using Chi-square test while Fischer exact test was used when expected cell count less than 5. Association between continuous variables measured for thyroid sparing and thyroid scarifying groups was tested using student T-test. The level of significance (p-value) was set at 0.005. Kaplen Meier statistics was used to measure survival.

## 3 Results

One hundred patients were included in this study (Table 1). Thyroidectomy was done in 40 patients; hemithyroidectomy in 38 and total thyroidectomy in two patients (thyroid sacrificing group). Thyroid gland was spared in 60 patients (thyroid sparing group). The study is parallel with allocation ratio (1.5). All the patients of the study (n = 100) were males. The mean age of the patients was 55 years in the thyroid sacrificing groups and 57 in the thyroid sparing group. Regarding the risk factors, all the patients (n = 100) were smokers and on the other hand, alcohol consumption was not reported in any patient.

**Table 1:** The baseline demographic and clinical characteristics for each group.

		Numbers		
		Thyroid sacrificing	Thyroid sparing	Total
Age		40–80	40–80	
Gender	Male	40	60	100
	Female	0	0	0
Tumor location	Glottic	12	21	33
	Supraglottic	10	14	24
	Subglottic	13	21	34
	Transglottic	29	40	69
T stage	T3	22	33	55
	T4	20	25	45
N stage	N0	36	53	89
	N1	2	1	3
	N2a	1	2	3
	N2b	1	1	2
	N2c	0	0	0
	N3	0	0	0
Tumor grade	Grade I	7	14	21
	Grade II	31	35	66
	Grade III	1	8	9
Tumor histological type	Squamous cell carcinoma			
	Other variants … Atypical spindle	1	1	2
	Basaloidscc	0	1	1
	Undifferentiated scc	0	1	1
True vocal fold mobility	Mobile	21	21	42
	Fixed	19	39	58
Local spread/invasion	Anterior commissure	6	2	8
	Laryngeal ventricle			
	Subglottic space			
	Pre-epiglottic space	8	10	
	Para-glottic space	7	8	15
	Pyriform sinus	6	13	19
	Thyroid cartilage	23	20	43
	Cricothyroid membrane			
	Cricoid cartilage	5	3	8
	Extra-laryngeal spread	8	12	20
	Thyroid gland	1	1	2
Preoperative radiotherapy		3	3	6
Neck dissection				
Thyroidectomy	Hemi thyroidectomy	40	0	40
	Total thyroidectomy	0	0	0
Postoperative radiotherapy		40	60	100
Recurrence	Nodal	1	3	4
	Stoma/peristomal	1	2	3
	Thyroid gland	0	1	1

The disease profiles of the two groups were similar regarding the location of the tumor, T stage (*p* = 0.181), N stage (*p* = 0.408), grade of differentiation (*p* = 0.281), histological variants of carcinoma (*p* = 0.281) and the need for adjuvant radiotherapy (*p* = 0.796).

Regarding the local tumor spread, the anterior commissure was involved in 15% of patients in the thyroid sacrificing group and in 3.3% in thyroid sparing group, which was statistically significant (*p* = 0.032). Similarly, thyroid cartilage invasion was noted in a significantly higher percentage of patients in thyroid sacrificing group (57.5%) than in thyroid sparing group (33.3%) (*p* = 0.017). On the other hand, there was no statistically significant difference between groups regarding spread or invasion to other subsites of the larynx (subglottis, preepiglottic space, paraglottic space, cricoid cartilage), or extralaryngeal spread (pyriform sinus, tongue base).

In the thyroid sacrificing group, infiltration of the thyroid gland by the laryngeal carcinoma was proved histopathologically in only 1 out of 40 patients (2.5%). In this patient the tumor was primary subglottic carcinoma. Cricoid cartilage destruction was detected with CT evaluation. However, there was no definite radiologic evidence of thyroid gland invasion before surgery. The clinical predictors of the thyroid gland invasion by laryngeal carcinoma could not be accurately assessed in this study due to the small number of patients, and gland invasion was detected in only one patient.

The incidence of postoperative hypothyroidism was significantly higher in the thyroid sacrificing group, where it was noticed in 25 out of 40 patients (62.5%), in contrast to only 4 out of 60 patients (6.7%) in the thyroid sparing group (*p* = 0.004). The incidence of hypothyroidism in relation to the type of thyroidectomy was as follows: hemithyroidectomy (23/38) and total thyroidectomy (2/2). The mean level of TSH among patients who developed hypothyroidism was 35.6 mIU/L (10.9–82.3 mIU/L). Thyroid hormone replacement was prescribed for all patients who developed hypothyroidism to alleviate clinical manifestations.

There was no statistically significant difference between the groups regarding the incidence of tumor recurrence. In the follow up period, two recurrences (5%) were detected in the thyroid sacrificing group; nodal recurrence (n = 1) and stomal recurrence (n = 1), and five recurrences (8.3%) in the thyroid sparing group; nodal recurrence (n = 3) and stomal recurrence (n = 2) (p = 0.408).

## 4 Discussion

In 1955, Ogura et al. [15] was the first to suggest en-bloc resection of the ipsilateral thyroid lobe and isthmus during laryngectomy. The rationale for thyroidectomy is that laryngeal tumors may spread to the gland either in a direct fashion through planes of least resistance, hematogenous or lymphatic spread [16]. However, routine thyroidectomy in locally advanced laryngeal carcinoma is still controversial. The adopted protocol in our center is that thyroidectomy is performed only in presence of subglottic extension, cricoid cartilage invasion or evidence of thyroid gland invasion. This is similar to previous studies [7,12] which recommended performing thyroidectomy in only selected cases in the presence of various predictive factors.

In this study thyroid gland invasion by laryngeal carcinoma was detected in only one patient in the thyroid sacrificing group. This patient had subglottic carcinoma with cricoid cartilage invasion. Previous studies have demonstrated that subglottic extension and invasion of the thyroid or cricoid cartilage is associated with tumor invasion into the thyroid gland [16,17]. However, in the present study, the clinical predictors of the thyroid gland invasion by laryngeal carcinoma could not be accurately assessed due to the small number of patients, and gland invasion was detected in only one patient.

The incidence of the thyroid gland invasion with a locally advanced laryngeal carcinoma is low. Mendelson et al. [6] and Kumar et al. [5], respectively, reported a pooled rate of TGI of 8% and 10.8% in two meta-analyses in 2009 and 2013. Similarly, Dogan et al. [7], Gorphe et al. [17] and Panda et al. [3] reported incidence of 2.3%, 12.6% and 8.8%, respectively. In our series, the incidence was 2.5%. These findings point to the unnecessary removal of thyroid gland in a high percentage of patients when routine thyroidectomy is performed with laryngectomy.

Furthermore, thyroid gland dysfunction after laryngectomy is a serious clinical condition. It was reported to occur much more frequently when performing thyroidectomy (hemi or total) with laryngectomy, than when performing laryngectomy only, with an incidence of 50–89% [[9], [18], [19], [20]]. In our study, hypothyroidism occurred in 62.5% of patients who underwent thyroidectomy and in 6.7% of patients who underwent laryngectomy without thyroidectomy. Thyroid dysfunction may lead to delayed wound healing, constipation and depression [10,11,[21], [22]]. It may therefore be beneficial to preserve the thyroid gland to prevent this morbidity and to improve the quality of life of patients.

Thyroid gland preservation did not seem to affect the oncological outcome or the survival rates in previous studies [1,3,6]. McGuire et al. [1] reported local recurrence rates of 6.8% (5/73) in the thyroid sparing group and 8.7% (6/69) in the thyroid lobectomy group (*p* = 0.76). Similarly, recurrence rates in our study were 5% (2/40) in the thyroid sparing group and 8.3% (5/60) in the thyroid sacrificing group, with no statistically significant differences between the two groups regarding local recurrence of disease (*p* = 0.408). This indicates that thyroid preservation in selected cases did not compromise oncologic control.

## 5 Conclusions

The incidence of thyroid gland invasion by an advanced laryngeal carcinoma is low. Preservation of the thyroid gland during laryngectomy to reduce the risk of thyroid dysfunction does not affect the oncological control. Thyroidectomy is however indicated if there is radiological or intraoperative evidence of extra-laryngeal tumor contacting or infiltrating the thyroid gland.

## Ethical approval

Faculty of medicine mansora university Institutional Research Board (MFM-IRB: MD/16.05.94).

## Sources of funding

No financial support has been received for this research.

## Author contributions

May El-Sebai : data collection.

Mahmoud Abd El-Shaheed: study design and data interpretation.

Hisham Ebada: data collection.

EL Sharawy Kamal: study design and data interpretation.

Ahmed Musaad: study design and data interpretation.

## Registration of research studies

1. Name of the registry: May Elsebai.

2. Unique Identifying number or registration ID: researchregistry7438.

3. Hyperlink to your specific registration (must be publicly accessible and will be checked): ttps://www.researchregistry.com/browse-the-registry#home/

## Guarantor

May Elsebai.

## Provenance and peer review

Not commissioned, externally peer-reviewed.

## Disclosure of interest

There is no conflict of interest or financial disclosure to be made.

## Data availability statement

The data supporting the findings of this study are available from the corresponding author upon reasonable request.

## Consent

Written consent was obtained and is available.

## Declaration of competing interest

There is no financial or personal relationships with other people or organisations for this research.
